# Depressive Symptoms and Health-Related Quality of Life Among Participants in the *Pasos Adelante* Chronic Disease Prevention and Control Program, Arizona, 2005-2008

**Published:** 2011-12-15

**Authors:** Christina A. Cutshaw, Lisa K. Staten, Kerstin Reinschmidt, Christopher Davidson, Denise J. Roe

**Affiliations:** Mel and Enid Zuckerman College of Public Health, University of Arizona; School of Medicine, Indiana University, Indianapolis, Indiana; Mel and Enid Zuckerman College of Public Health, University of Arizona, Tucson, Arizona; Mel and Enid Zuckerman College of Public Health, University of Arizona, Tucson, Arizona; Mel and Enid Zuckerman College of Public Health, University of Arizona, Tucson, Arizona

## Abstract

**Introduction:**

Chronic diseases are the leading causes of death in the United States and have been associated with depressive symptoms and poor health-related quality of life (HRQOL). This study examined whether depressive symptoms and HRQOL indicators changed among participants in *Pasos Adelante,* a chronic disease prevention and control program implemented in a US–Mexico border community.

**Methods:**

*Pasos Adelante* was a 12-week *promotora*-led program that included educational sessions and walking groups. We used the Centers for Epidemiologic Studies Depression Scale (CES-D) and the Center for Disease Control's "Healthy Days" measures to measure depressive symptoms and HRQOL. We used linear mixed-effects models and general estimating equations to analyze changes in CES-D scores and HRQOL indicators from baseline to postprogram and from postprogram to 3-month follow-up.

**Results:**

At baseline, participants had a mean of 7.1 physically unhealthy days, 7.4 mentally unhealthy days, and 3.9 days of activity limitation. The mean number of physically and mentally unhealthy days declined significantly from baseline to postprogram, but the mean number of activity limitation days did not. At baseline, 42.6% of participants reported their health as fair/poor; 20.8% of participants reported frequent mental distress, and 31.8% had a CES-D score of 16 or more. All 3 proportions declined from baseline to postprogram. No significant changes occurred between postprogram and follow-up.

**Conclusion:**

Participants in *Pasos Adelante* showed improvement in depressive symptoms and several HRQOL indicators. Future studies should use an experimental design with a comparison group to determine whether these findings can be replicated and to examine potential mediators and moderators of program effects.

## Introduction

In the United States, chronic diseases are the leading causes of death (1) and are responsible for most health care costs (2). People with chronic conditions such as asthma, arthritis, coronary artery disease, stroke, and diabetes are also more likely to have poor mental health, such as depression, than people without these chronic conditions (3). One reason for comorbid chronic disease and depression is that chronic disease risk factors such as smoking, physical inactivity, obesity, and heavy alcohol consumption are more common among people with a diagnosis of depression, symptoms of depression, or frequent mental distress (ie, 2 weeks or more in the last month that mental health was not good) (4-6).

Chronic disease also negatively affects health-related quality of life (HRQOL) (7,8), or "perceived physical and mental health over time" (8). In a national study of 6,000 people with 6 types of chronic conditions, only one-fourth reported their overall health was excellent or very good compared to more than one-half of participants in a survey of the general population (9). Having multiple chronic conditions rather than none or fewer conditions is associated with worse HRQOL in multiple domains (10).

Among Hispanic people, the largest ethnic minority in the United States (11), 4 of the 5 leading causes of death are chronic diseases (1). The public health community is interested in changing factors, such as poor diet and physical inactivity, that can increase the risk of developing cardiovascular disease or diabetes or complicate these conditions if they are already present (12). Community-based lifestyle-intervention programs facilitated by *promotores* *de salud* (community health workers) show promise for reducing chronic disease risk factors among Hispanic populations (13,14). Highly structured, long-term (eg, 5-12 months) lifestyle-intervention programs that focus on weight and physical activity have improved depressive symptoms and HRQOL for women with polycystic ovary disease (15) and improved HRQOL among postmenopausal women with type 2 diabetes (16). It is not known, however, whether shorter, less structured community-based lifestyle-intervention programs can change depressive symptoms and HRQOL among a primarily female Hispanic population aged 50 years or older. The objective of this study was to examine whether depressive symptoms and HRQOL indicators changed over time among participants in *Pasos Adelante,* a 12-week chronic disease prevention and control program implemented in a US–Mexico border community. We hypothesized that participants in *Pasos Adelante* would show improvements in depressive symptoms and HRQOL.

## Methods

### Study design

The *Pasos Adelante* program was 1 of 3 chronic disease prevention and control programs conducted in Douglas, Arizona, by the Prevention Research Center at the Mel and Enid Zuckerman College of Public Health at the University of Arizona. The purpose of the *Pasos Adelante* program was to reduce chronic disease risk factors among participants. As part of the program's evaluation, we established 2 sets of endpoints. Primary endpoints for *Pasos Adelante* are reported elsewhere (17) and included body mass index, waist and hip circumference, waist-hip ratio, systolic and diastolic blood pressure, pulse, total cholesterol, triglycerides, high-density lipoprotein, low-density lipoprotein, and blood glucose. Secondary endpoints, the focus of this study, were changes in depressive symptoms and HRQOL. We used a quasi-experimental within-subjects design with assessments at baseline, immediately after the program ended, and approximately 3 months postprogram to examine these secondary endpoints. All study protocols were approved by the University of Arizona institutional review board, and we obtained informed consent from participants.

### Study setting and recruitment

Details on the Douglas community are described elsewhere (17). The study included ten 12-week rounds (program periods). In any given round, 1 or more groups of participants participated in the program. Recruitment for *Pasos Adelante* took place between January 2005 and February 2008; *promotoras* used a variety of convenience methods to recruit participants, including in-person recruitment at local public events (eg, health fairs, Rotary Club meetings). Participants also provided referrals. Individuals could participate more than once in the program, but we included data for this study from each participant'sinitial participation only. Inclusion criteria for the study included being aged 18 years or older and residing in the Douglas community. A total of 327 participants enrolled in the program. We excluded 22 participants from our analyses because they participated in other Prevention Research Center programs. The total number of participants included in our analyses was 305.

### The *Pasos Adelante* program

The educational component of the program consisted of group sessions led by 2 *promotoras* that met once per week in community settings, beginning in January 2008 and ending in August 2008. We adapted the curriculum from the first edition of *Su Corazon, Su Vida* (Your Heart, Your Life) by the National Heart, Lung and Blood Institute (http://hp2010.nhlbihin.net/salud/pa/indexsp.htm), which addressed such topics as risk factors for heart disease; physical activity; high blood pressure, salt and sodium; dietary fats and cholesterol; healthy cooking and eating; and smoking cessation. Adaptations for *Pasos Adelante* included information about diabetes, blood glucose and its relationship with dietary sugar, and community health assessment (http://azprc.arizona.edu/resources/curricula). In rounds 6 through 10, we also included information on ways to reduce stress and depression, which was drawn from a curriculum about diabetes and depression (18).

Physical activity was an integral part of the *Pasos Adelante* program. The program encouraged participants to walk or exercise on their own, and each group session included a walk or other physical activity. *Promotoras* walked with participants at scheduled times at the beginning of the program but gradually decreased their participation until they were no longer walking with the group. The program encouraged participants to continue the group walks and to take responsibility for scheduling them and reminding other participants to attend. *Promotoras* also called participants to remind them about classes and walking groups. Participants received a certificate, canvas bag, water bottle, and diabetes educational materials after the final session.

We designed the *Pasos Adelante* program on the basis of behavioral science theories and ethnic and cultural considerations. We integrated social cognitive or social learning theory (19) into the program by including personal goal-setting exercises and encouraging participants to monitor their own progress by reviewing weekly goals. Our belief in the importance of social support in initiating and maintaining healthy behaviors guided the choice of delivering the program in a group setting. We also designed the program to reflect the ecological model, which emphasizes multiple levels of influence on behavior (19), by including curriculum materials that acknowledged family, cultural, and economic influences on food purchases and preparation choices and that educated participants on how to use advocacy to make their communities healthier. Finally, we conducted the sessions and assessments in Spanish, the preferred language of the participants, and used local *promotoras* as facilitators to make the program as attentive as possible to cultural and community norms. More details on the curriculum and its development are available elsewhere (14).

### Procedures

At baseline for each participant, *promotoras* and other program staff conducted an interview, took anthropometric measurements, and made referrals for a fasting blood draw. Baseline was defined as the point at which participants completed informed consent and the first interview. Postprogram assessment took place from immediately after session 12 through 6 weeks later. Follow-up assessment took place approximately 12 to 18 weeks after session 12. At the postprogram and follow-up assessments, 2 *promotoras* performed the anthropometric and physiologic measures, and trained university staff conducted the interviews. No one conducting postprogram and follow-up assessments had access to baseline data. Details on these procedures are available elsewhere (17).

### Measures

During the baseline interview, we assessed demographic and health characteristics using a pencil-and-paper 70-item questionnaire administered in either English or Spanish (17). We used the same questionnaire with minor word changes in the social support section at postprogram and follow-up assessment. The questionnaire included these domains in the following order: demographic characteristics, life priorities, physical activity, dietary practices, social support, HRQOL, medical history, access to medical care and insurance, clinical tests received in the previous 6 months, the Centers for Epidemiologic Studies Depression Scale (CES-D), and use of alcohol and tobacco. The questionnaire took about 45 minutes (range, 20-90 min) to complete. Of the 305 participants, all completed the baseline assessment, 255 (83.6%) completed the postprogram assessment, 221 (72.5%) completed the follow-up assessment, and 217 (71.1%) completed all 3 assessments.

We used the Centers for Disease Control and Prevention's HRQOL-4, or "Healthy Days" measures, the same 4 questions used by the Behavioral Risk Factor Surveillance System (20), to measure HRQOL. For self-rated health, the question is, "Would you say that in general your health is excellent, very good, good, fair, or poor"? For physically unhealthy days, the question is, "Now thinking about your physical health, which includes physical illness and injury, for how many days during the past 30 days was your physical health not good?" For mentally unhealthy days, the question is, "Now thinking about your mental health, which includes stress, depression, and problems with emotions, for how many days during the past 30 days was your mental health not good?" For activity limitation days, the question is, "During the past 30 days for about how many days did poor physical or mental health keep you from doing your usual activities, such as self-care, work, or recreation?" We asked about activity-limitation days only if the participant reported any physically or mentally unhealthy days. Response options for each of these measures are 0 to 30 days. The Healthy Days measures have been used in many studies (21) and have established validity and reliability (22).

We dichotomized self-rated health responses into "fair/poor" or "good/very good/excellent" as other studies have done (10). Because we were interested in mental health indicators, we also used a variable for frequent mental distress (FMD); we coded the number of mentally unhealthy days into 2 dichotomous values (0 to 13 days and 14 or more days) and used 14 or more days to indicate FMD. The FMD variable has been used in studies by itself (20) and along with the number of mentally unhealthy days (23).

We measured depressive symptoms with the 20-item self-report CES-D (24). The CES-D has been used in clinical and population studies (25) and with Hispanic populations (26), including Mexican Americans (27). Symptoms are scored according to frequency in the previous week: less than 1 day (score = 0), 1 or 2 days (score = 1), 3 or 4 days (score = 2), and 5 to 7 days (score = 3) (21); total response scores are created by summing all 20 responses after reverse-coding 4 questions (questions 4, 8, 12, and 16). We considered a score of 16 or more in this study to indicate distress that may have reached a clinical level because other studies have used this threshold (27). The CES-D has high internal consistency (Cronbach α = 0.85 for community samples and 0.90 for clinic samples) and moderate test-retest reliability (*r *= 0.45-0.70) (24).

### Statistical analysis

We summarized baseline demographic variables by using the number and percentage for categorical variables and the mean and standard deviation (SD) for continuous variables. We constructed linear mixed-effects models to analyze the effect of time on the change in means for the continuous HRQOL measures (total number of physically unhealthy days, total number of mentally unhealthy days, and total number of activity-limitation days) from baseline to postprogram and from postprogram to follow-up. For self-rated health, FMD, and CES-D scores, we used general estimating equations (GEE) to analyze the effect of time on the odds ratios for changing categories from baseline to postprogram and from postprogram to follow-up. Both the linear mixed-effects and GEE models included all 305 participants with at least 1 assessment. We used SAS software version 9.2 (SAS Institute Inc, Cary, North Carolina) to conduct all analyses.

## Results

Characteristics of the participants (n = 305) and differences between the 217 participants that completed the program (ie, had 3 assessments) and the 88 participants that did not are described elsewhere (17). Most participants (n = 207) completed at least 9 of the 12 educational classes.

At baseline, participants on average had 7.1 physically unhealthy days, 7.4 mentally unhealthy days, and 3.9 days of activity limitation (Table 1). Overall, the mean number of physically and mentally unhealthy days declined significantly from baseline to follow-up, but the mean number of activity limitation days did not (Table 2). The number of physically and mentally unhealthy days declined significantly from baseline to postprogram but not from postprogram to follow-up. The number of activity limitation days did not change, remaining at about 3 days from baseline through follow-up.

At baseline, 42.6% of participants reported their overall health as fair or poor (Table 1); 20.8% of participants reported FMD, and 31.8% had a CES-D score of 16 or more. The likelihood of reporting fair or poor health (vs excellent, very good, or good), reporting FMD (vs ≤13 mentally health days), or having a CES-D score of 16 or more (versus a score <16) at postprogram was significantly less than at baseline. We found no differences from postprogram to follow-up.

## Discussion

Participants in *Pasos Adelante* showed improvements in depressive symptoms and several HRQOL indicators from baseline to 3-month follow-up. Participants maintained improvements from postprogram to follow-up. The improvement in self-rated health is particularly promising because self-rated health has been consistently identified as a predictor of mortality even when controlling for health conditions and other confounding variables (27). One possible explanation for the lack of change in limited activity days is that the *Pasos Adelante* program, which is an educational program, did not provide treatment for physical or mental health problems; thus it may not have been able to address physical health or mental health needs severe enough to limit activity. Future studies should more carefully assess activity limitations in a population with physical and mental health problems.

Although *Pasos Adelante* was not a treatment program, it was able to effect significant change in a sample with a high rate of self-reported fair or poor health (42.6%) and a high rate of FMD (20.8%) at baseline. To put our findings in context, in a community sample of 44,649 Hispanic women in the 1993-2001 Behavioral Risk Factor Surveillance System (BRFSS), the prevalence of fair or poor self-rated health was 24.2%, and the prevalence of FMD was 10.6% for women and 10.5% for Hispanics (28).

This study has limitations. It did not have a comparison group, so we cannot rule out possible explanations (eg, attention from the *promotoras*) for our observations. An experimental study, perhaps a randomized controlled trial, is needed to better understand the program's effect. Future research also needs to explore potential mediators, such as changes in weight and physical activity, and moderators, such as baseline levels of HRQOL and depressive symptoms, on the outcomes to better understand how the program may affect mental health and perceived mental and physical health (ie, HRQOL) and for whom the program may work best (29).

The promising effect of *Pasos Adelante* on depressive symptoms and HRQOL suggests that a *promotora*-led community-based chronic disease prevention and control program focused on changing behavioral risk factors through education and encouragement of physical activity can improve dimensions of HRQOL and depressive symptoms among participants in a primarily Hispanic border community. Our findings, combined with significant improvements in anthropometric and clinical outcomes (17), suggest that with future study, *Pasos Adelante* may be a useful component in strategies to address 2 primary *Healthy People 2020* goals: eliminating racial/ethnic disparities in chronic disease and increasing HRQOL (30).

## Figures and Tables

**Table 1.** Health-Related Quality-of-Life (HRQL) Indicators and Depressive Symptoms at Baseline, Postprogram, and 3-Month Follow-Up Among Participants (N = 305) in a Chronic Disease Prevention and Control Program, Arizona, 2005-2008

Measure	Baseline			Postprogram			Follow-Up		

	No. of Respondents	Mean (SD)	Median (IQR)	No. of Respondents	Mean (SD)	Median (IQR)	No. of Respondents	Mean (SD)	Median (IQR)
Physically unhealthy days	304	7.1 (10.1)	2.0 (0.0-10.0)	255	5.2 (8.4)	1.0 (0.0-7.0)	221	5.7 (8.9)	2.0 (0.0-7.0)
Mentally unhealthy days	303	7.4 (10.1)	3.0 (0.0-10.0)	255	4.8 (8.0)	2.0 (0.0-6.0)	221	4.7 (8.1)	1.0 (0.0-5.0)
Activity limitation days^a^	230	3.9 (6.5)	0.0 (0.0-7.0)	175	3.8 (6.6)	0.0 (0.0-5.0)	150	3.6 (6.7)	0.0 (0.0-4.0)

**Measure**	**No. of Respondents**	**n (%)**		**No. of Respondents**	**n (%)**		**No. of Respondents**	**n (%)**	
Has fair or poor self-rated health	305	130 (42.6)		255	85 (33.3)		221	82 (37.1)	
Has frequent mental distress (≥14 mentally unhealthy days)	303	63 (20.8)		255	27 (10.6)		221	25 (11.3)	
CES-D score ≥16^b^	305	97 (31.8)		255	63 (24.7)		221	57 (25.8)	

Abbreviations: SD, standard deviation; IQR, interquartile range; CES-D, Centers for Epidemiologic Studies Depression Scale.

a We asked about activity limitation days only if participant reported any physically or mentally unhealthy days. Data were missing for this variable.

b Symptoms are scored according to frequency in the previous week: less than 1 day (score = 0), 1 or 2 days (score = 1), 3 or 4 days (score = 2), and 5 to 7 days (score = 3) (22); total response scores are created by summing all 20 responses. We considered a score of 16 or more to indicate distress that may have reached a clinical level (28).

**Table 2.** Changes in Health-Related Quality-of-Life Indicators and Depressive Symptoms Among Participants (n = 305) in a Chronic Disease Prevention and Control Program, Arizona, 2005-2008

Measure	Baseline to Follow-Up, Overall Effect of Time	Baseline to Postprogram		Postprogram to Follow-Up	

	Linear Mixed-Effects Models				

	*P* Value	Estimate (95% CI)	*P*	Estimate (95% CI)	*P*
Physically unhealthy days	.02	−1.78 (−3.04 to −0.52)	.006	0.50 (−0.86 to 1.87)	.47
Mentally unhealthy days	<.001	−2.50 (−3.63 to −1.29)	<.001	−0.24 (−1.50 to 1.01)	.70
Activity limitation days^a^	.85	−0.10 (−1.32 to 1.12)	.88	−0.28 (−1.63 to 1.08)	.69

**Measure**	**General Estimating Equations**				

	***P* Value**	**OR (95% CI)**	***P***	**OR (95% CI)**	***P***

Has fair or poor self-rated health	.007	0.70 (0.56 to 0.88)	.002	1.15 (0.89 to 1.50)	.29
Has frequent mental distress (≥14 mentally unhealthy days)	<.001	0.53 (0.36 to 0.78)	.002	1.01 (0.65 to 1.57)	.97
CES-D score ≥16^b^	.04	0.73 (0.55 to 0.96)	.03	1.04 (0.77 to 1.39)	.81

Abbreviations: OR, odds ratio, CI, confidence interval; CES-D, Centers for Epidemiologic Studies Depression Scale.

a We asked about activity limitation days only if participant reported any physically or mentally unhealthy days. Data were missing for this variable.

b Symptoms are scored according to frequency in the previous week: less than 1 day (score = 0), 1 or 2 days (score = 1), 3 or 4 days (score = 2), and 5 to 7 days (score = 3) (22); total response scores are created by summing all 20 responses. We considered a score of 16 or more to indicate distress that may have reached a clinical level (28).
